# Supplementary material for: A Dihydropyridine Derivative as a Highly Selective Fluorometric Probe for Quantification of Au3+ Residue in Gold Nanoparticle Solution
Source: Sensors (Basel). 2022 Dec 30;23(1):436. doi: 10.3390/s23010436 (PMC9823494; doi:10.3390/s23010436)
Supplement: Supplementary file 1 [file sensors-23-00436-s001.zip › sensors-2077004-supplementary.pdf]

Supporting Information

for

A Dihydropyridine Derivative as a Highly Selective Fluorometric Probe for Quantification of Au^{3+} Residue in Gold Nanoparticle Solution

Waroton Paisuwan [1], Mongkol Sukwattanasinitt [2], Mamoru Tobisu [3,4] and Anawat Ajavakom [2,*]

1 Department of Chemistry, Faculty of Science, Chulalongkorn University, Phyathai Road, Bangkok 10330, Thailand
2 Nanotec-CU Center of Excellence on Food and Agriculture, Department of Chemistry, Faculty of Science, Chulalongkorn University, Phyathai Road, Bangkok 10330, Thailand
3 Department of Applied Chemistry, Graduate School of Engineering, Osaka University, Osaka 565-0871, Japan
4 Innovative Catalysis Science Division, Institute for Open and Transdisciplinary Research Initiatives (ICS-OTRI), Osaka University, Osaka 565-0871, Japan
* Correspondence: anawat.a@chula.ac.th

TABLE OF CONTENTS	PAGE
S1. ^{1}H NMR spectra of DHP in acetone-d_6	2
S2. ^{1}H NMR spectra of DHP-OH in acetone-d_6	2
S3. ^{13}C NMR spectra of DHP in acetone-d_6^{1}H	3
S4. ^{13}C NMR spectra of DHP-OH in acetone-d_6	3
S5. HRMS spectra of DHP	4
S6. HRMS spectra of DHP-OH	4
S7. IR spectra of DHP	5
S8. IR spectra of DHP-OH	5
S9. Molar absorptivity of DHP and DHP-OH	6
S10. Fluorescence quantum efficiency of DHP and DHP-OH	7
S11. Fluorescence response of DHP and [DHP+Au^{3+}] in various pHs	8
S12. Fluorescence response of DHP-OH and [DHP-OH+Au^{3+}] in various pHs	8
S13. Time dependent fluorescence quenching profile in Tris-HCl pH 6.0 for 60 minutes	9
S14. Comparison of fluorescent probes for Au^{3+} detection	9-10
S15. Absorption spectra of DHP-OH, Au^{3+}, and the mixture of DHP-OH and Au^{3+} in Tris-HCl pH 6.0.	10
S16. ^{13}C NMR spectra of [DHP-OH+Au^{3+}]	11
S17. HRMS spectra of [DHP-OH+Au^{3+}]	11
S18. Absorption and morphology of AuNPs	12

S1. ^{1}H NMR spectra of DHP in acetone-d_6

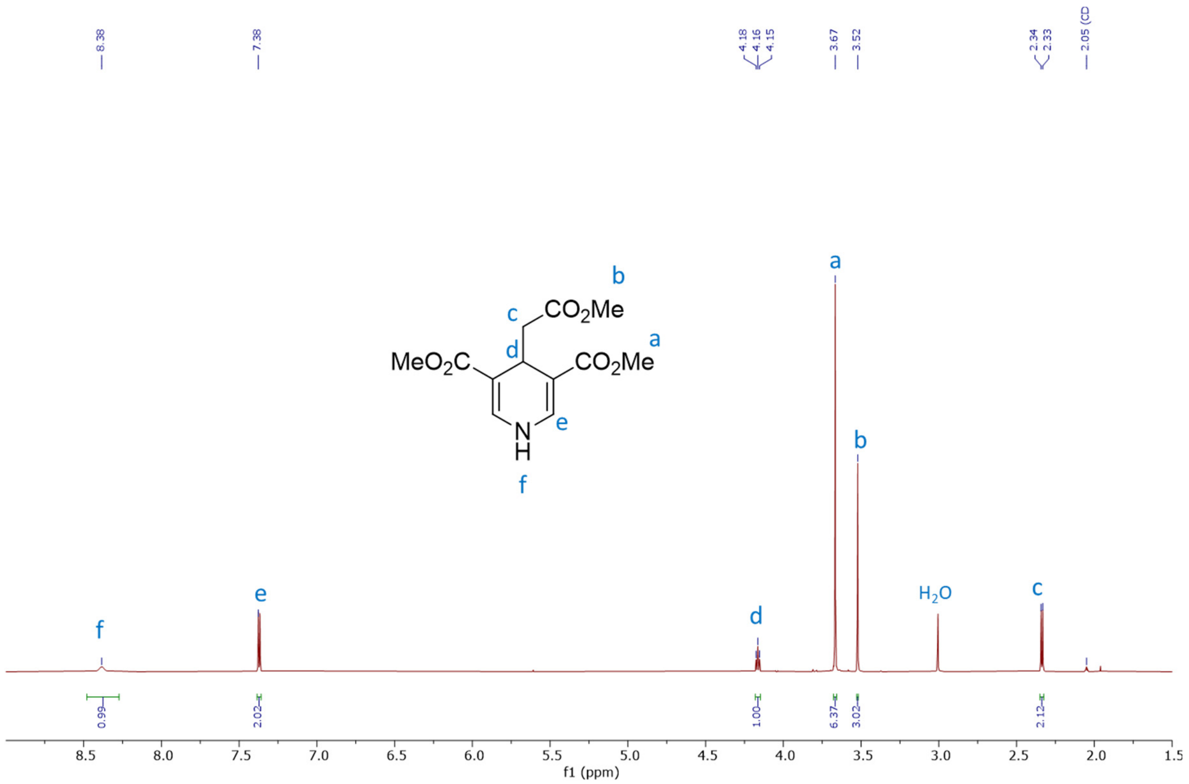

S2. ^{1}H NMR spectra of DHP-OH in acetone-d_6

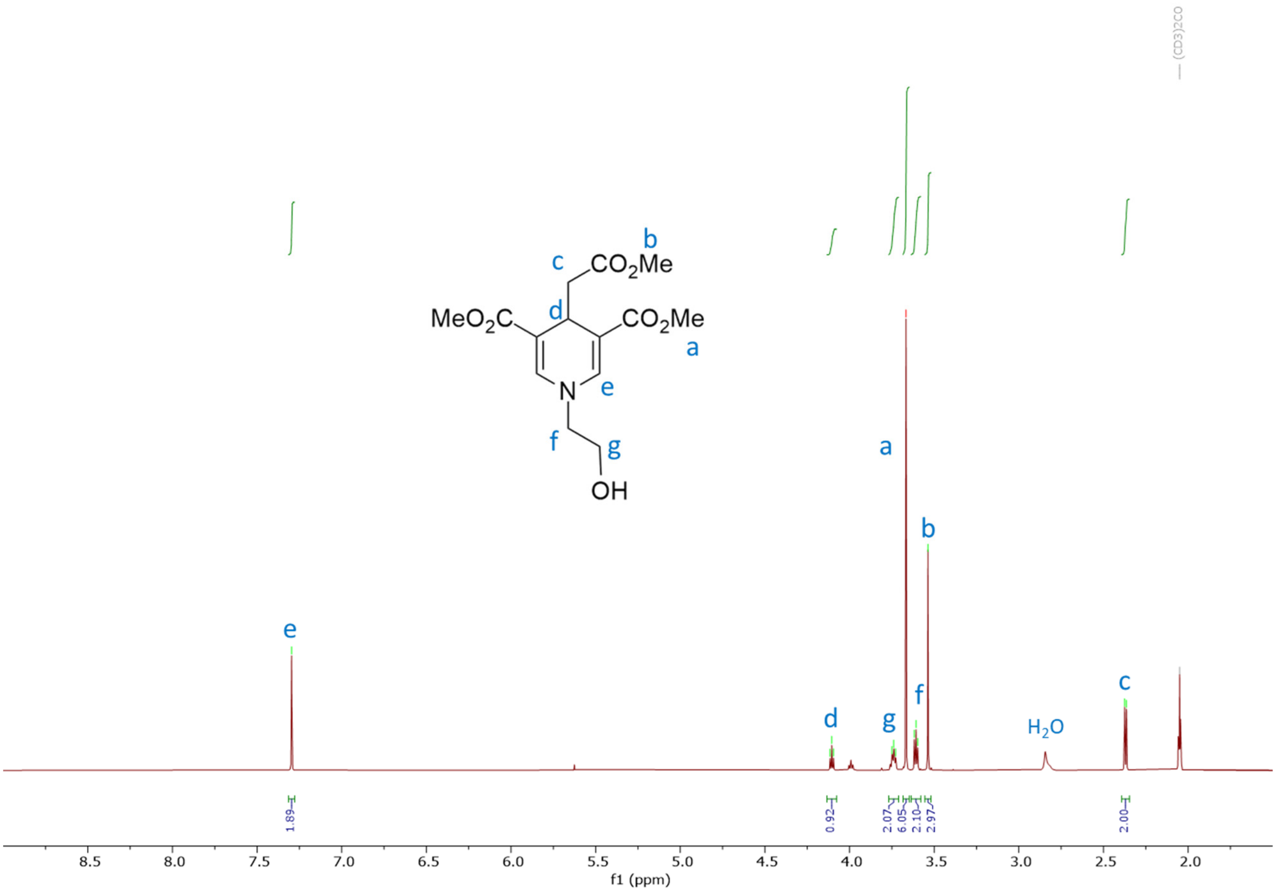

S3. ^{13}C NMR spectra of DHP in acetone-*d₆*

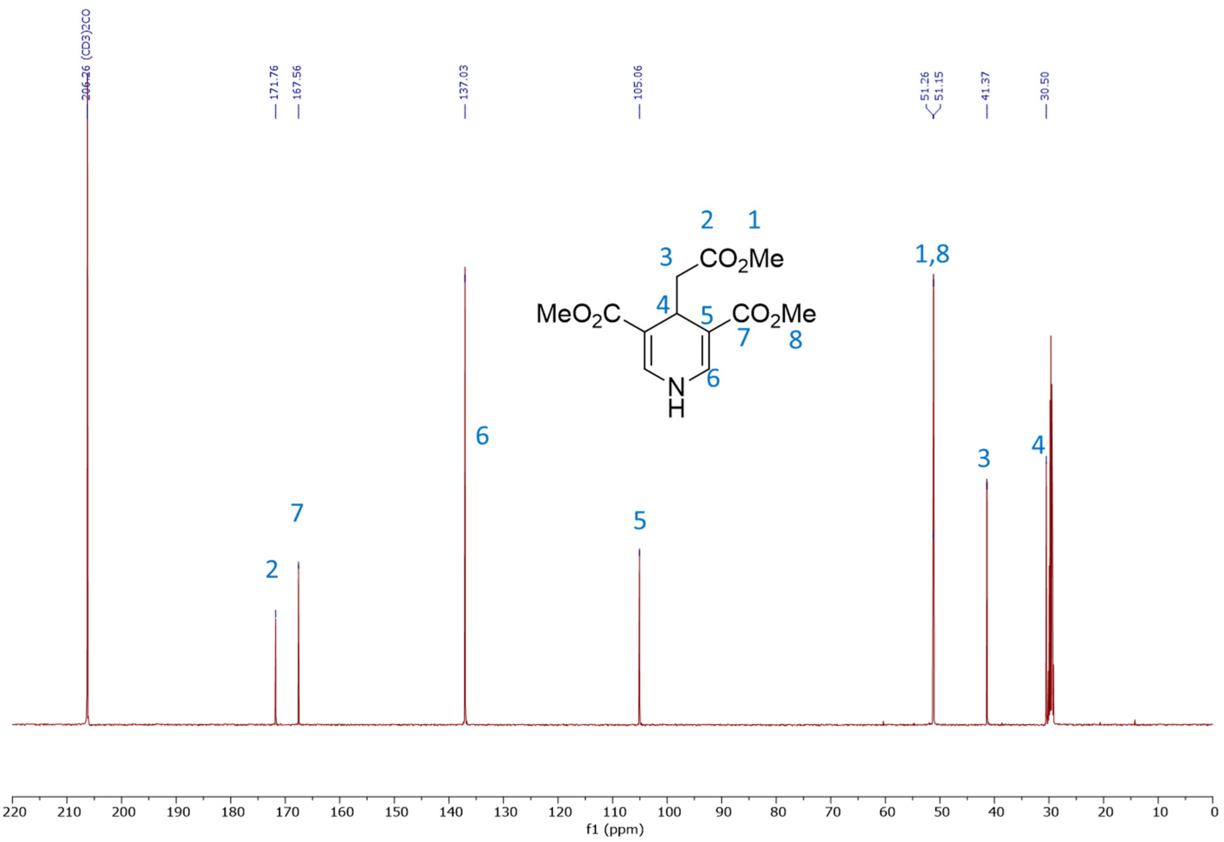

S4. ^{13}C NMR spectra of DHP-OH in acetone-*d₆*

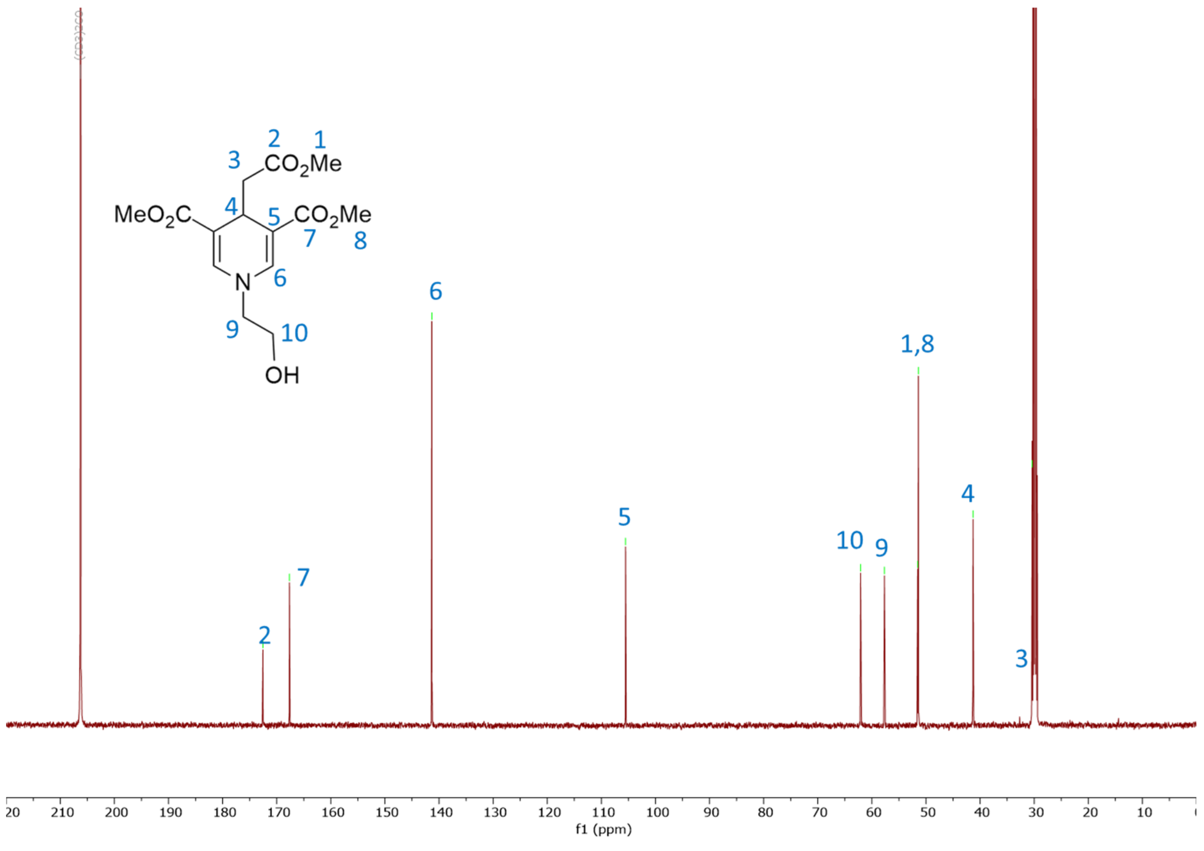

S5. HRMS spectra of DHP

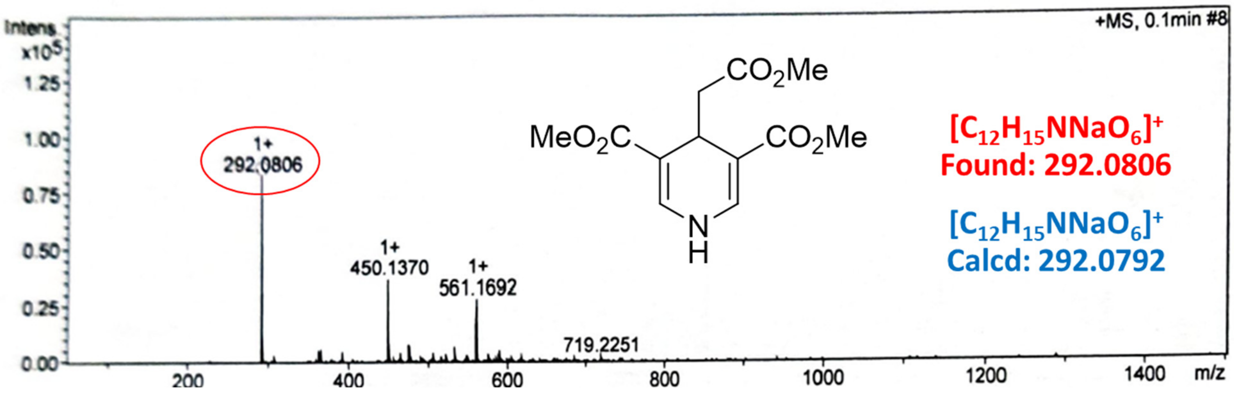

S6. HRMS spectra of DHP-OH

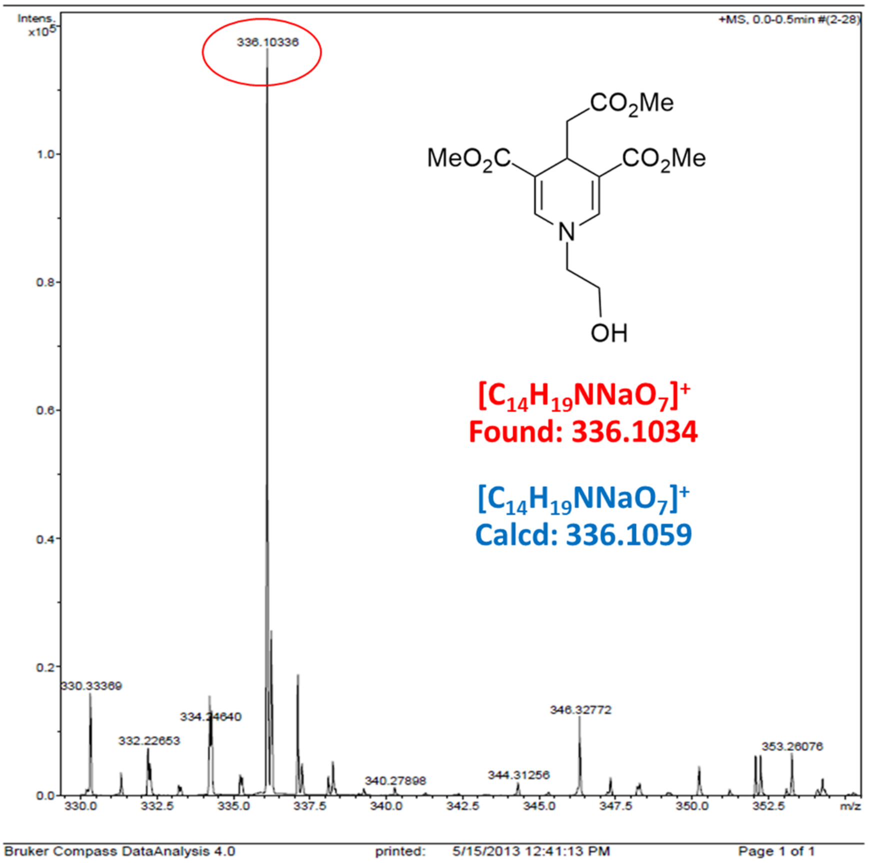

S7. IR spectra of DHP

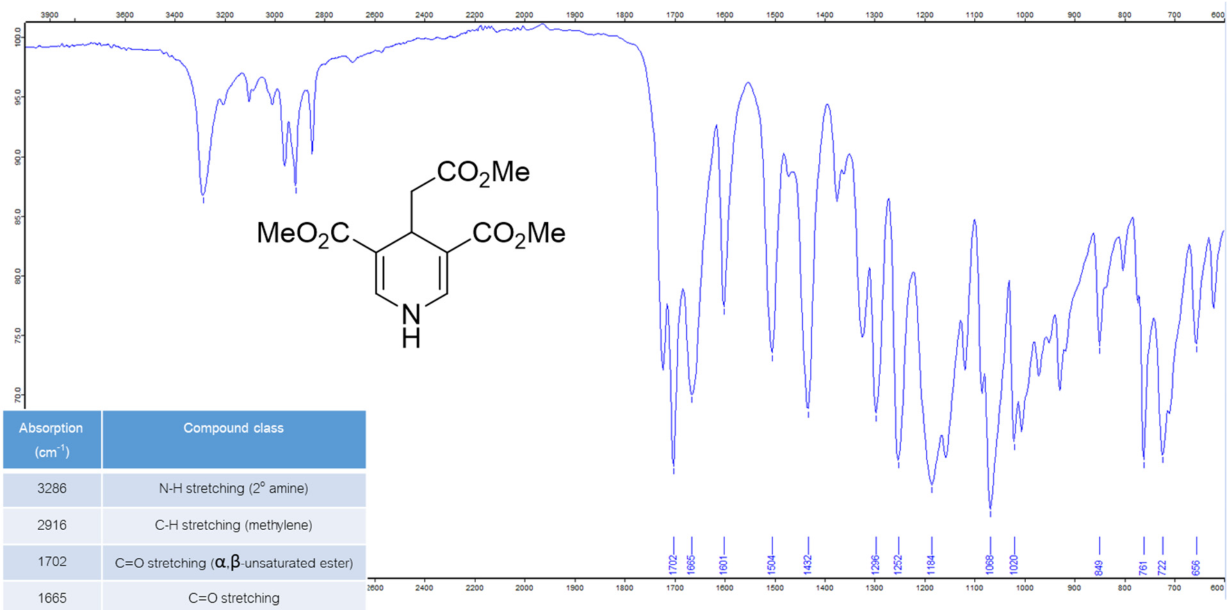

Absorption (cm^{-1})	Compound class
3286	N-H stretching (2° amine)
2916	C-H stretching (methylene)
1702	C=O stretching (α,β-unsaturated ester)
1665	C=O stretching
1601	C=C stretching (cyclic alkene)
1504	N-H bending
1432	C-H bending (alkane)
1296	O-H bending
1252	C-N stretching (amine)
1184	C-O stretching (ester)

S8. IR spectra of DHP-OH

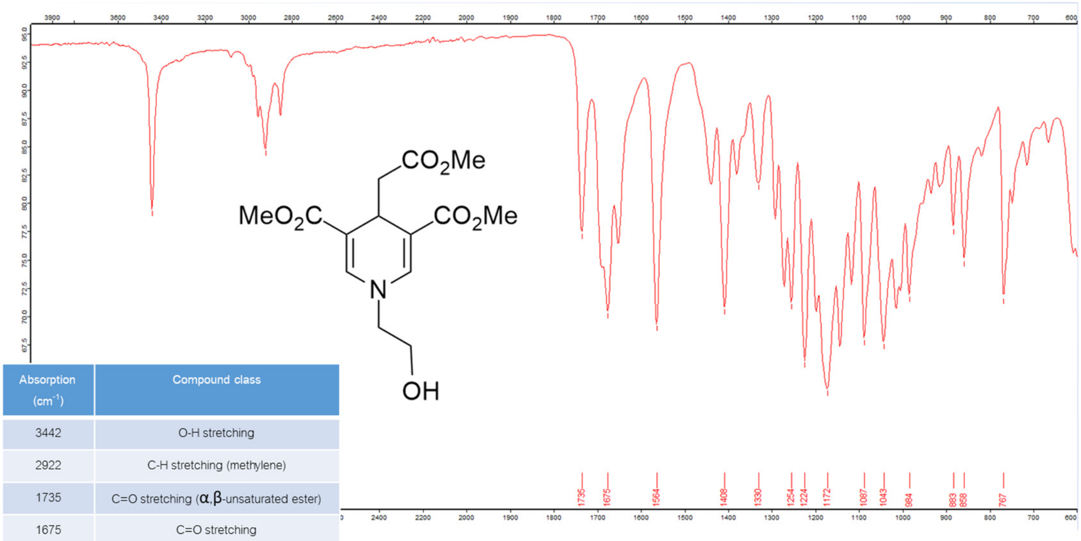

Absorption (cm^{-1})	Compound class
3442	O-H stretching
2922	C-H stretching (methylene)
1735	C=O stretching (α,β-unsaturated ester)
1675	C=O stretching
1564	C=C stretching (cyclic alkene)
1408	C-H bending (alkane)
1330	O-H bending
1254	C-N stretching (amine)
1172	C-O stretching (ester)
1087	C-O stretching (1° alcohol)

S9. Molar absorptivity of DHP and DHP-OH

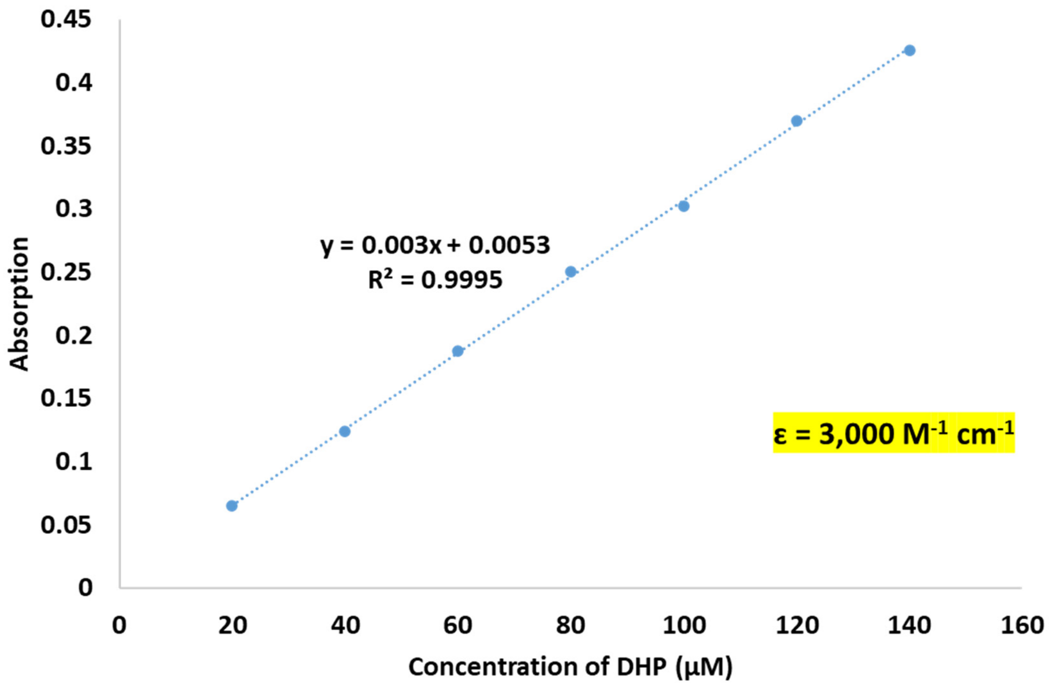

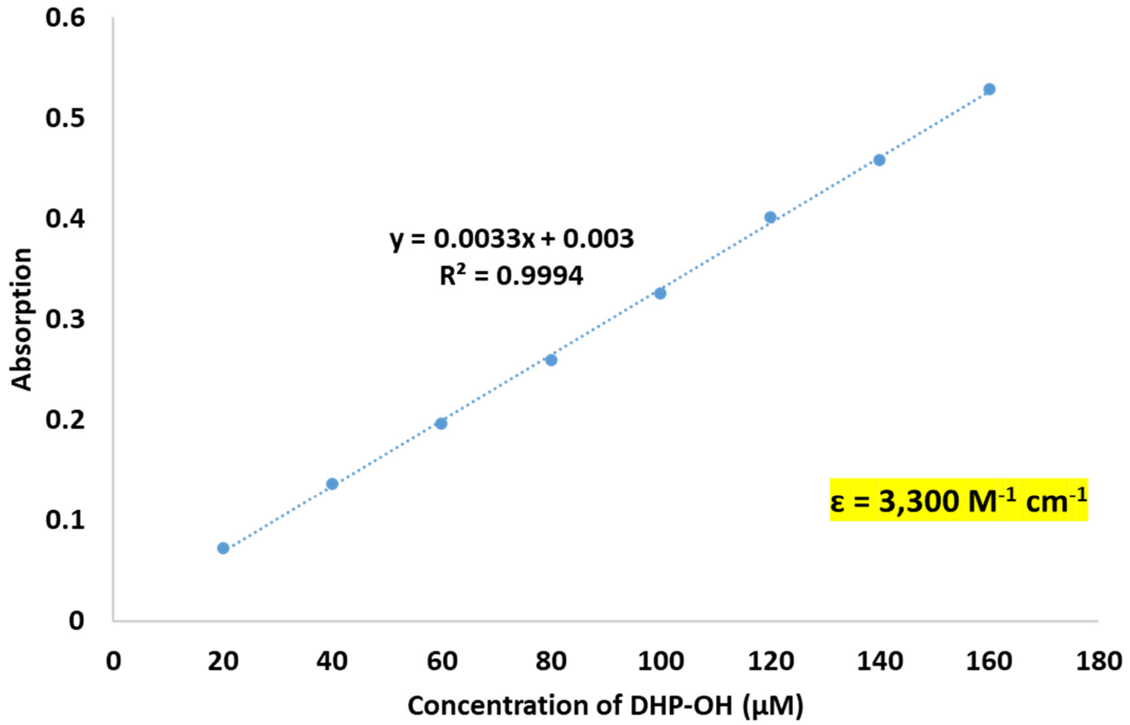

S10. Fluorescence quantum efficiency of DHP and DHP-OH

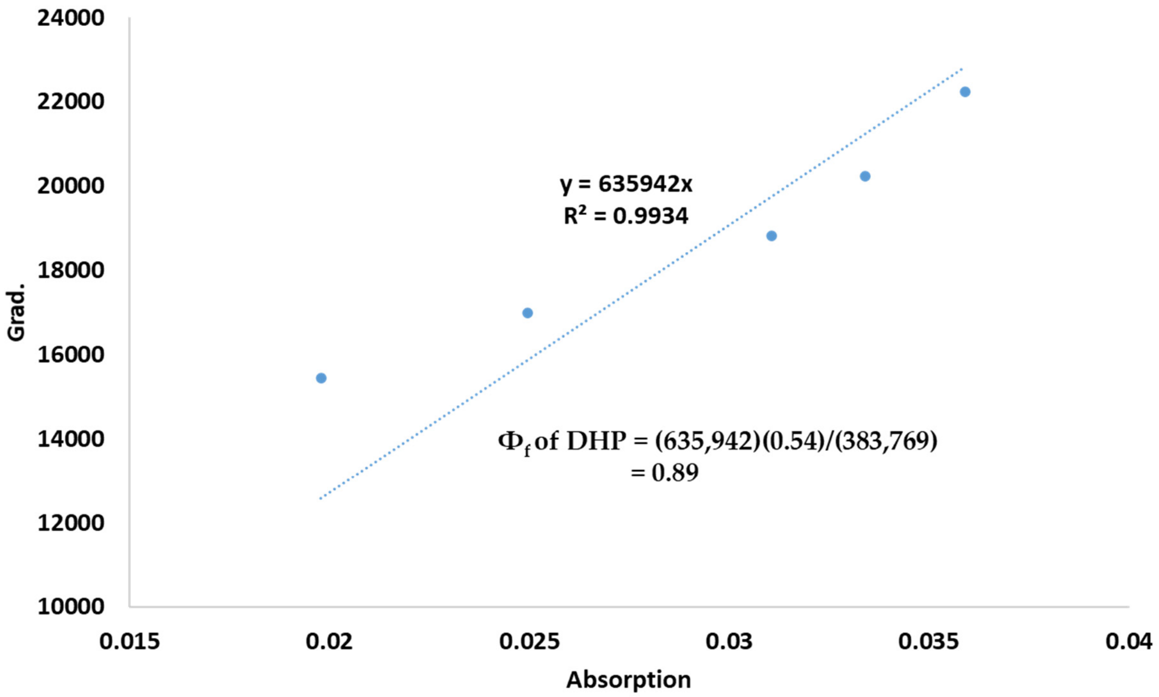

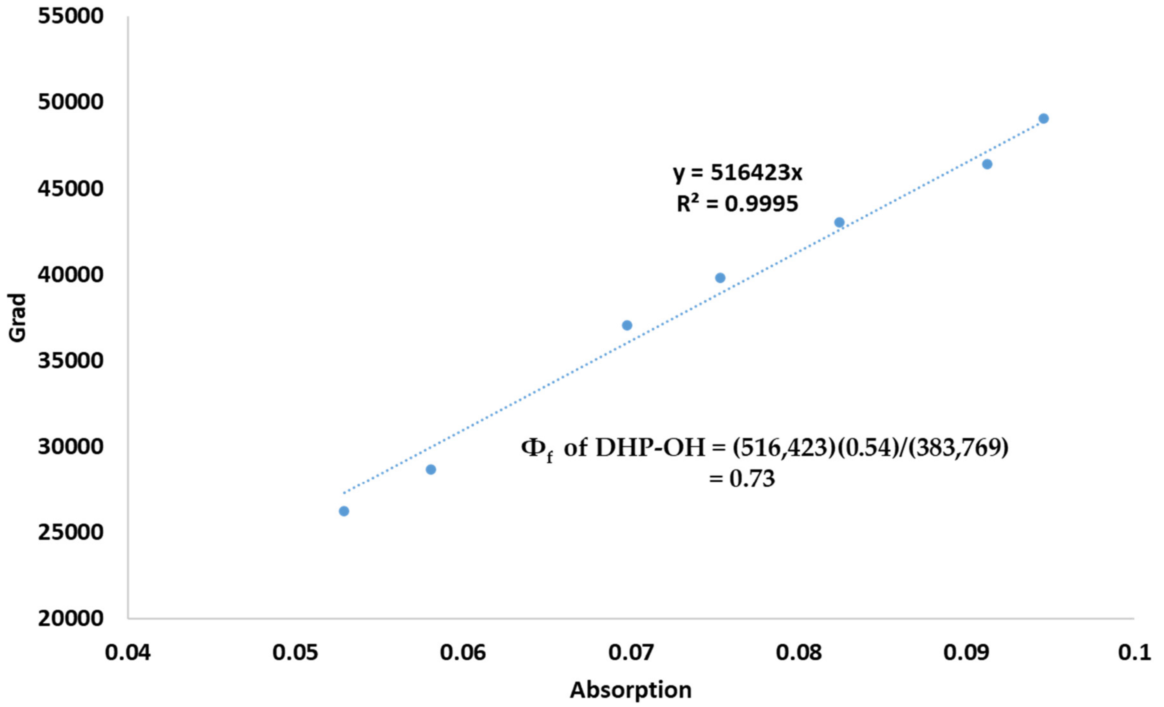

S11. Fluorescence response of DHP and [DHP+Au^{3+}] in various pHs

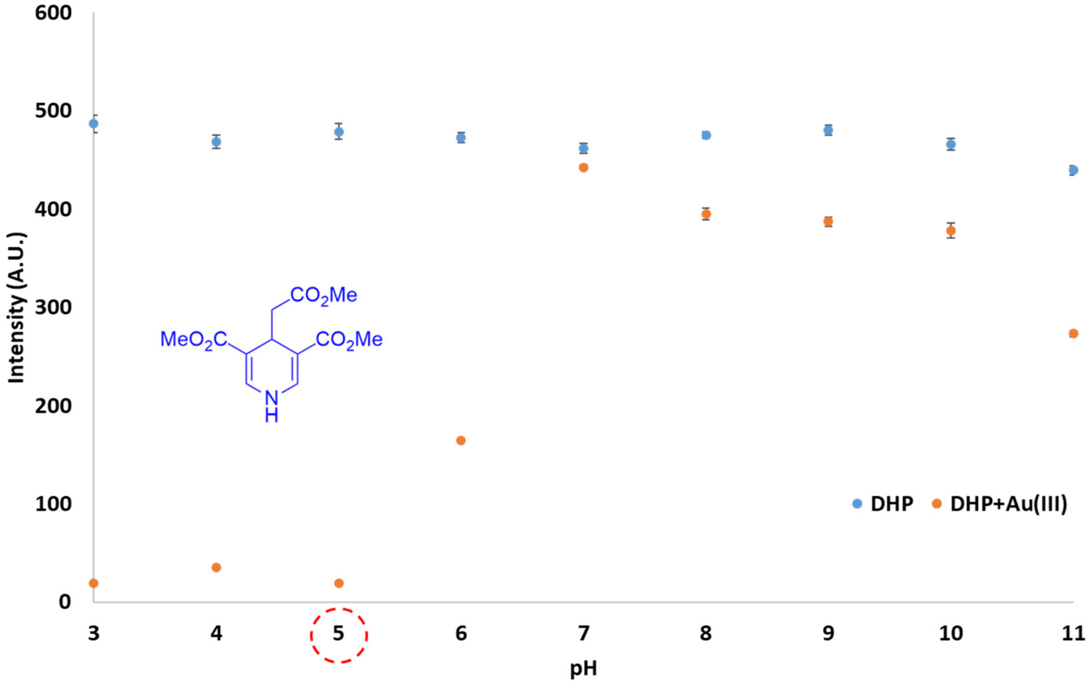

S12. Fluorescence response of DHP-OH and [DHP-OH+Au^{3+}] in various pHs

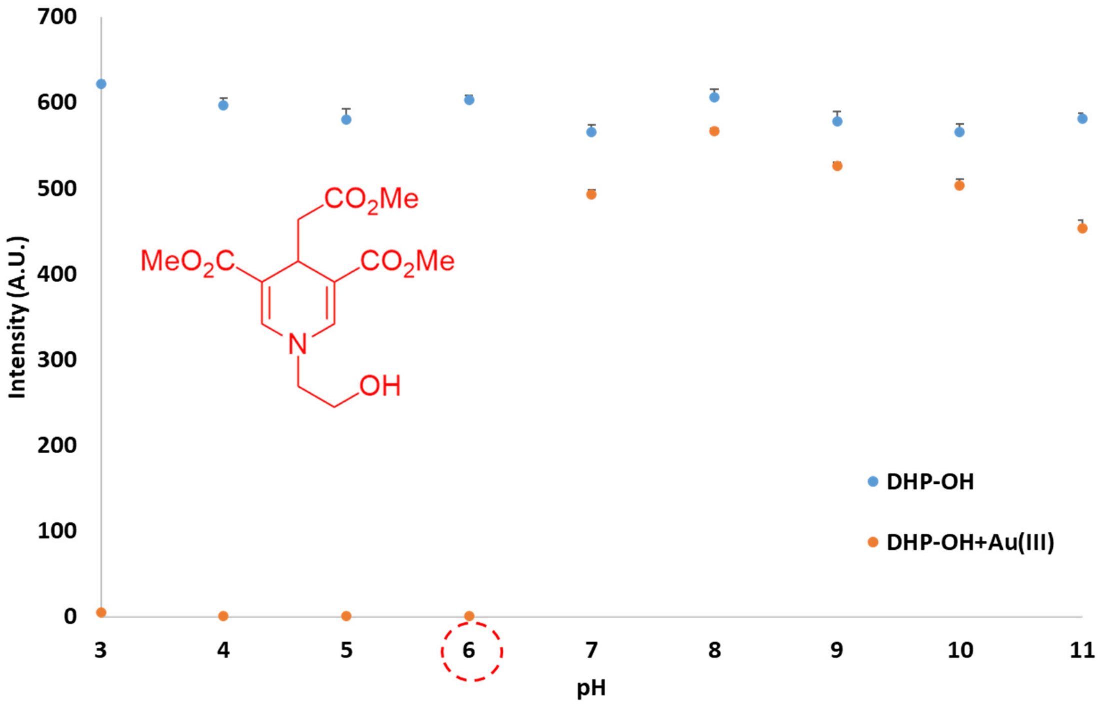

S13. Time dependent fluorescence quenching profile in Tris-HCl pH 6.0 for 60 minutes

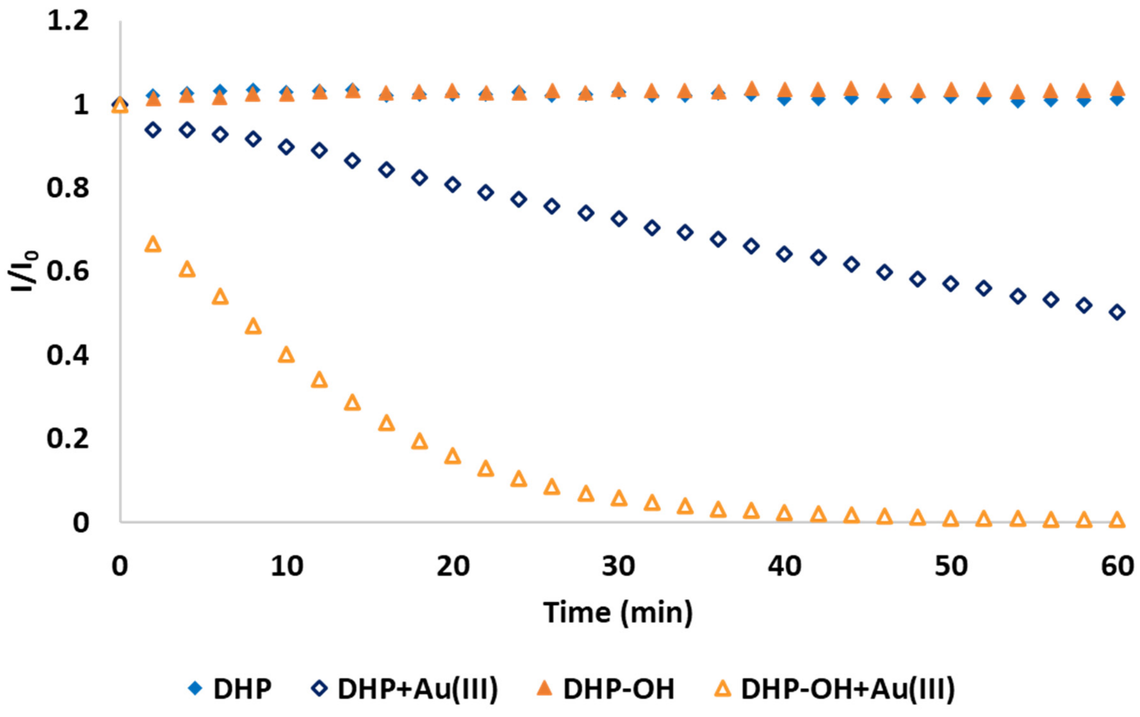

S14. Comparison of fluorescent probes for Au^{3+} detection

Entry	Fluorescent Probe	Synthetic step	LOD (nM)	Fluorescent Mode	Solvent (v/v)	Application	References
1	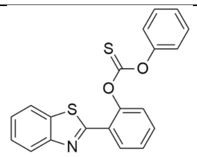	1	48	Turn on	DMSO/ H_2O (1/1)	Synthetic waste water	*Dyes and Pigments*, **2019**, 164, 14-19.
2	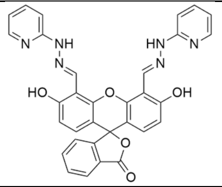	1	70	Turn on	HEPES (pH 7.4)	Cell imaging	*Sensors and Actuators B*, **2015**, 209, 1005-1010.
3	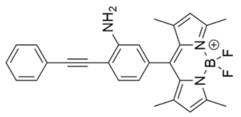	3	100	Turn on	EtOH/ PBS (1/1, pH 7.4)	Human blood, serum and cell imaging	*Sensors and Actuators B*, **2016**, 226, 364-369.
4	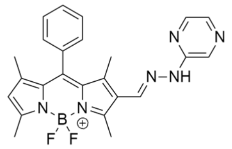	1	60	Turn on	PBS/ EtOH (7:3, pH 7.0)	Cell imaging	*Biosensors and Bioelectronics*, **2016**, 77, 812-817.
5	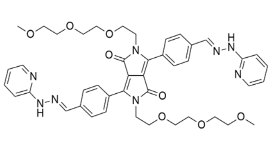	4	15	Turn on and ratiometric	EtOH/ PBS (1/1, pH 7.4)	Cell imaging	*Biosensors and Bioelectronics*, **2016**, 80, 288-293.

6	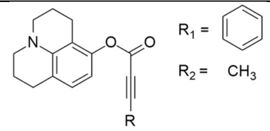	1	R_1=100 R_2=120	Turn on	EtOH/ HEPES (1/1, pH 7.4)	Cell imaging	*Biosensors and Bioelectronics*, **2016**, 86, 939-943.
7	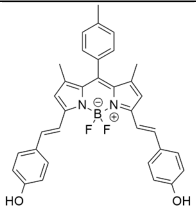	1	9.4	Turn on	MeOH/ H_2O (8:2)	Tap water and cell imaging	*Dyes and Pigments*, **2021**, 191, 109341.
8	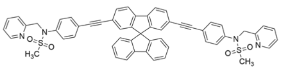	2	9.8	Turn off	DMSO/ NaCl-HCl (1/99, pH 2.0)	Drinking, rain, tap, pond, and river water	*Journal of Photochemistry and Photobiology A: Chemistry*, **2020**, 112823,
9	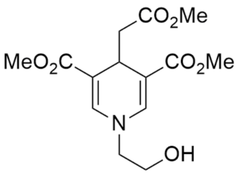	1	167 (32.8 ppb)	Turn off	Tris-HCl pH 6.0	Strip test, Real water samples, and Au^{3+} residue in AuNPs solution	**This work**

S15. Absorption spectra of DHP-OH, Au^{3+}, and the mixture of DHP-OH and Au^{3+} in Tris-HCl pH 6.0.

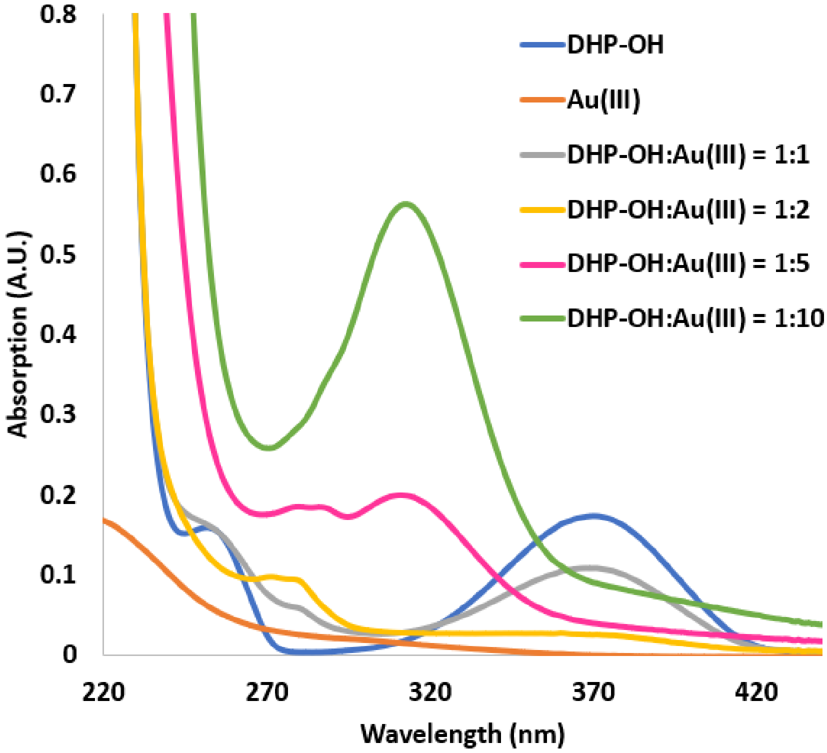

S16. ^{13}C NMR spectra of [DHP-OH+Au^{3+}]

1-(2-hydroxyethyl)-4-(2-methoxy-2-oxoethyl)-3,5-bis(methoxycarbonyl)pyridin-1-ium (Oxidized product): ^{13}C NMR (125 MHz, D_2O:DMSO-d_6 (99:1, v/v)) δ (ppm): 171.29, 163.23, 155.09, 148.76, 131.93, 64.03, 59.87, 54.11, 53.16, 37.99.

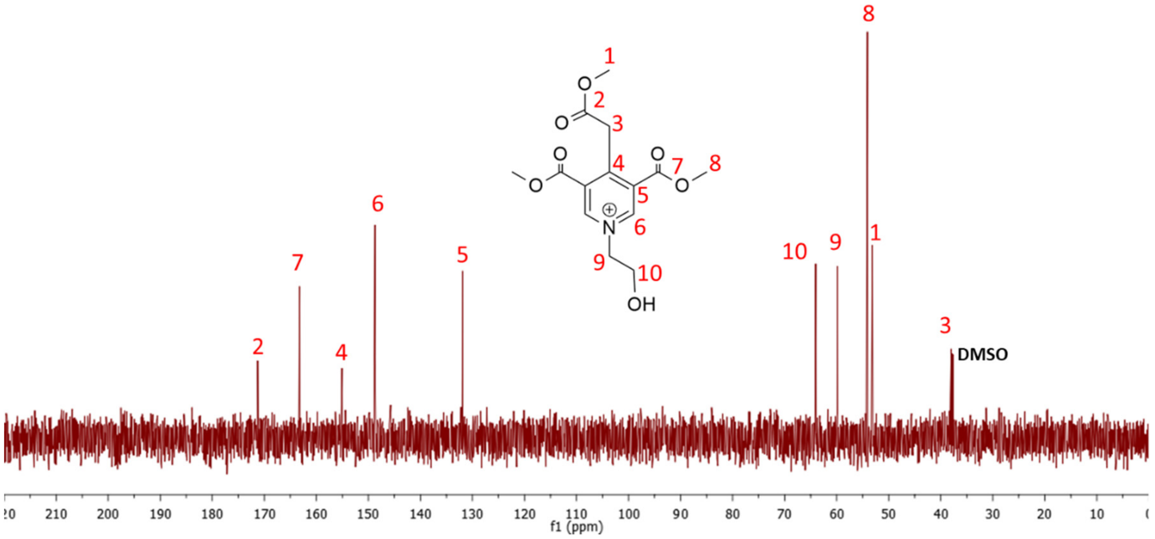

S17. HRMS spectra of [DHP-OH+Au^{3+}]

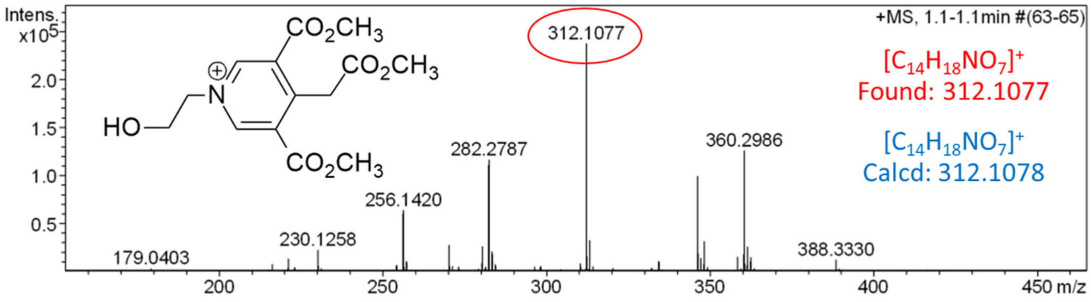

S18. Absorption and morphology of AuNPs

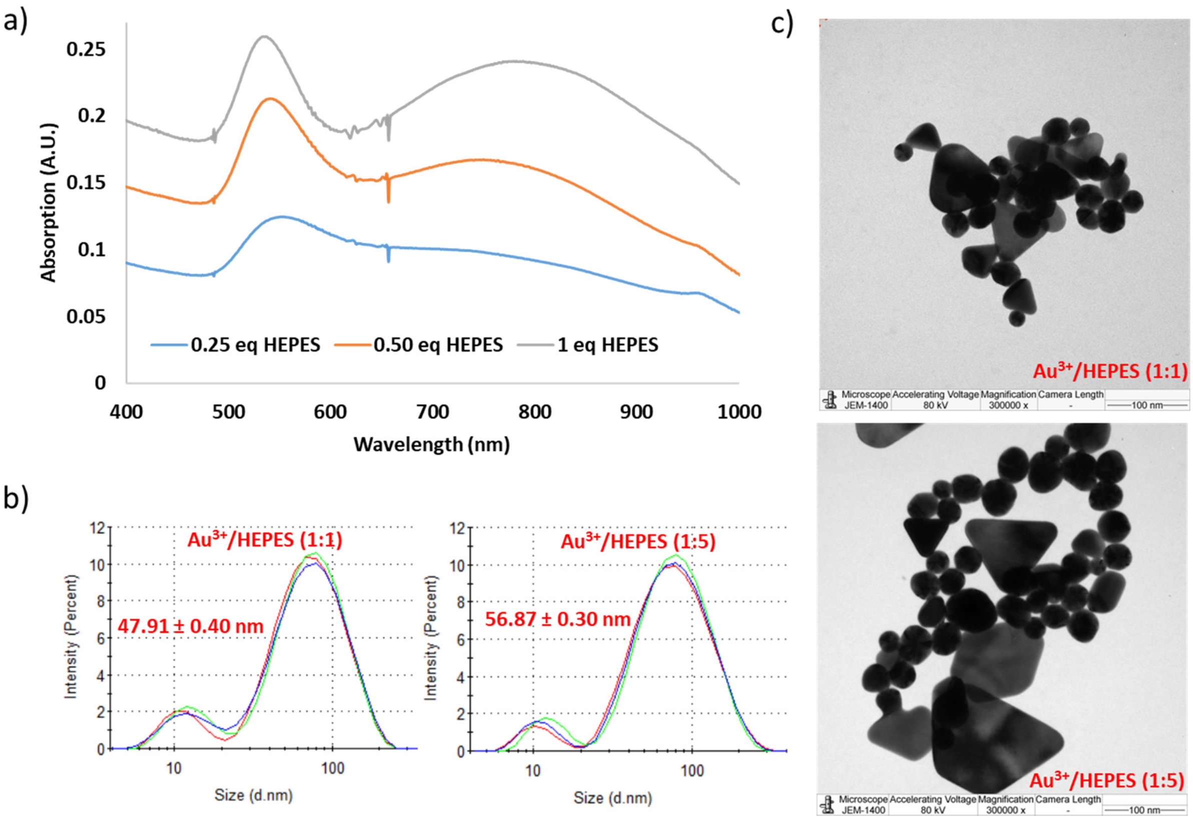

a) absorption spectra, b) DLS spectra, and c) TEM images of the Au^{3+}/HEPES solution at the molar ratio of 1:1 and 1:5.
